# Supplementary material for: Antibacterial Activity of Two Zn-MOFs Containing a Tricarboxylate Linker
Source: Nanomaterials (Basel). 2022 Nov 23;12(23):4139. doi: 10.3390/nano12234139 (PMC9736432; doi:10.3390/nano12234139)
Supplement: Supplementary file 1 [file nanomaterials-12-04139-s001.zip › nanomaterials-2005824-supplementary.pdf]

Supporting information

Antibacterial Activity of Two Zn-MOFs Containing a Tricarboxylate Linker

Sara Rojas [1], Amalia García-García [1], Tania Hidalgo [2], María Rosales [1], Daniel Ruiz-Camino [2], Pablo Salcedo-Abraira [2], Helena Montes-Andrés [1], Duane Choquesillo-Lazarte [3], Roberto Rosal [4], Patricia Horcajada [2,*] and Antonio Rodríguez-Diéguez [1,*]

[1] Department of Inorganic Chemistry, Faculty of Science, University of Granada, Av. Fuentenueva s/n, 18071 Granada, Spain
[2] Advanced Porous Materials Unit, IMDEA Energy Institute, Av. Ramón de la Sagra 3, 28935 Móstoles, Spain
[3] Laboratorio de Estudios Cristalográficos, IACT-CSIC, Av. de las Palmeras, 4, 18100 Armilla, Spain
[4] Department of Chemical Engineering, University of Alcalá, 28871 Alcalá de Henares, Spain
* Correspondence: patricia.horcajada@imdea.org (P.H.); antonio5@ugr.es (A.R.-D.)

Table of content

S1. Coordination modes……………………………………………………………………………..S2
S2. Crystallographic information……………………………………………………………………S3-S5
S3. Physicochemical characterization ……………………………………………………………S6-S8
S4. Antibacterial activity……………………………………………………………………………..S9-S10

S1. Coordination modes

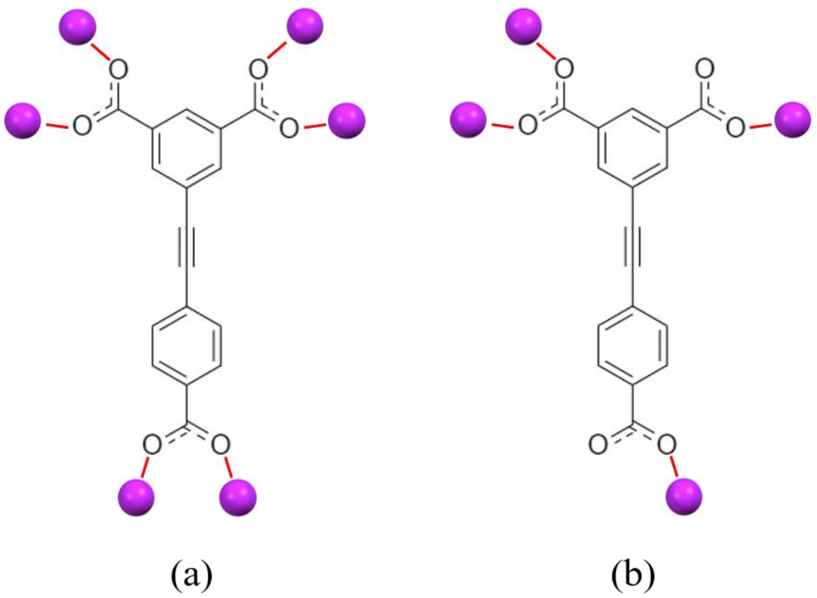

Figure S1. Coordination modes of the linker H_3L in the new MOFs. GR-MOF-8 contains modes (a) and (b), whereas GR-MOF-9 is made of linkers with coordination mode (a). Metal ions are indicated in purple color.

S2. Crystallographic information

Table S1. Table of the selected bond lengths (Å) and angles (°) for GR-MOF-8.

Bond	Distance (Å)	Angle	Degree (°)
Zn1-O1	1.985(10)	O1-Zn1-O22	104.6(5)
Zn1-O22	1.995(11)	O1-Zn1-O24	111.7(4)
Zn1-O24	1.931(9)	O1-Zn1-O25	102.7(6)
Zn1-O25	1.947(14)	O22-Zn1-O24	110.7(4)
Zn2-O8	1.967(9)	O22-Zn1-O25	114.2(5)
Zn2-O23	2.000(9)	O24-Zn1-O25	112.4(5)
Zn2-O24	1.969(8)	O8-Zn2-O23	101.7(4)
Zn2-O28	1.951(9)	O8-Zn2-O24	108.3(3)
Zn3-O2	1.974(11)	O8-Zn2-O28	109.6(4)
Zn3-O9	1.910(10)	O23-Zn2-O24	110.9(4)
Zn3-O24	1.943(7)	O23-Zn2-O28	103.9(4)
Zn3-O36	1.931(10)	O24-Zn2-O28	120.8(4)
Zn4-O24	1.929(9)	O2-Zn3-O9	107.0(5)
Zn4-O27	2.011(11)	O2-Zn3-O24	108.9(4)
Zn4-O35	2.008(9)	O2-Zn3-O36	107.4(5)
Zn4-O49	1.971(8)	O9-Zn3-O24	111.6(4)
		O9-Zn3-O36	112.5(4)
		O24-Zn3-O36	109.2(4)
		O24-Zn4-O27	108.6(5)
		O24-Zn4-O35	112.3(4)
		O24-Zn4-O49	121.9(4)
		O27-Zn4-O35	100.9(5)
		O27-Zn4-O49	110.2(4)
		O35-Zn4-O49	100.8(4)
		Zn1-O24-Zn2	108.1(4)
		Zn1-O24-Zn3	110.1(4)
		Zn1-O24-Zn4	107.7(4)
		Zn2-O24-Zn3	108.7(4)
		Zn2-O24-Zn4	112.0(4)
		Zn3-O24-Zn4	110.2(4)

Table S2. Table of the selected bond lengths (Å) and angles (°) for GR-MOF-9.

Bond	Distance (Å)	Angle	Degree (°)
Zn00-O005	1.97(2)	O005-Zn00-O009	100.7(11)
Zn00-O009	2.14(3)	O005-Zn00-O00B	100.6(11)
Zn00-O00B	2.13(3)	O005-Zn00-O0AA	105.8(11)
Zn00-O1	1.92(7)	O005-Zn00-O1A	108(2)
Zn00-O0AA	2.00(3)	O009-Zn00-O1A	151(2)
Zn00-O1A	2.14(6)	O00B-Zn00-O009	86.2(11)
Zn1-O005	1.95(3)	O00B-Zn00-O1A	91.2(18)
Zn1-O00E	2.04(3)	O1-Zn00-O005	104.9(19)
Zn1-O00T	1.96(6)	O1-Zn00-O009	153(2)
Zn1-O3	1.94(3)	O1-Zn00-O00B	80(2)
Zn1-O8CA	2.33(6)	O1-Zn00-O0AA	97.0(17)
Zn2-O005	1.96(3)	O0AA-Zn00-O009	84.6(11)
Zn2-O00D	2.05(3)	O0AA-Zn00-O00B	153.2(11)
Zn2-O00J	1.93(3)	O0AA-Zn00-O1A	84.9(19)
Zn2-O01X	1.94(6)	O005-Zn1-O00E	108.5(11)
Zn2-O0CA	2.33(6)	O005-Zn1-O00T	121(2)
Zn04-O005	2.00(3)	O005-Zn1-O8CA	83.6(15)
Zn04-O00K	2.05(6)	O00E-Zn1-O8CA	153.7(17)
Zn04-O2	2.07(6)	O00T-Zn1-O00E	100.7(19)
Zn04-O1AA	2.05(6)	O00T-Zn1-O8CA	54(2)
Zn04-O2AA	2.04(6)	O3-Zn1-O005	113.0(11)
Zn04-O3AA	1.97(3)	O3-Zn1-O00E	100.7(13)
		O3-Zn1-O00T	111(2)
		O3-Zn1-O8CA	95.4(18)
		O005-Zn2-O00D	108.0(11)
		O005-Zn2-O0CA	83.5(15)
		O00D-Zn2-O0CA	155.0(16)
		O00J-Zn2-O005	112.7(11)
		O00J-Zn2-O00D	100.8(13)
		O00J-Zn2-O01X	112(2)
		O00J-Zn2-O0CA	94.6(17)
		O01X-Zn2-O005	120(2)
		O01X-Zn2-O00D	101.1(18)
		O01X-Zn2-O0CA	55(2)
		O005-Zn04-O00K	94.6(18)
		O005-Zn04-O2	94.4(19)
		O005-Zn04-O1AA	94.5(18)
		O005-Zn04-O2AA	95(2)
		O1AA-Zn04-O2	171(3)
		O2AA-Zn04-O00K	170(3)
		O3AA-Zn04-O005	177.6(12)

	O3AA-Zn04-O00K	84.4(19)
	O3AA-Zn04-O2	87.7(19)
	O3AA-Zn04-O1AA	83.6(19)
	O3AA-Zn04-O2AA	86(2)
	Zn00-O005-Zn1	109.2(12)
	Zn00-O005-Zn2	108.7(12)
	Zn00-O005-Zn04	107.9(11)
	Zn1-O005-Zn2	110.8(11)
	Zn1-O005-Zn04	110.3(12)
	Zn2-O005-Zn04	109.9(12)

S3. Physicochemical characterization

The lattice parameters were refined using TOPAS software (version 5, Bruker AXS, Karlsruhe, Germany). There is a good agreement between the data and the model.

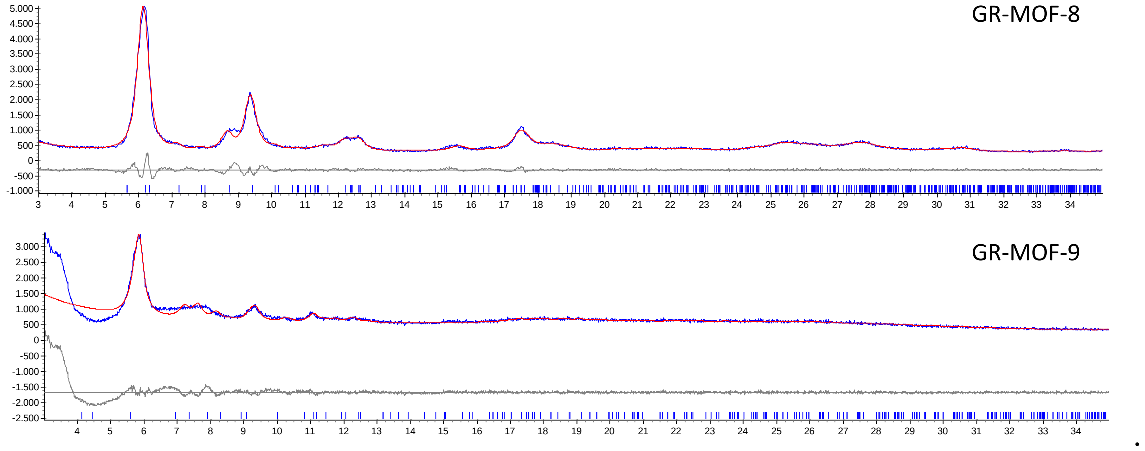

Figure S2. Le Bail fitting of GR-MOF-8 and 9.

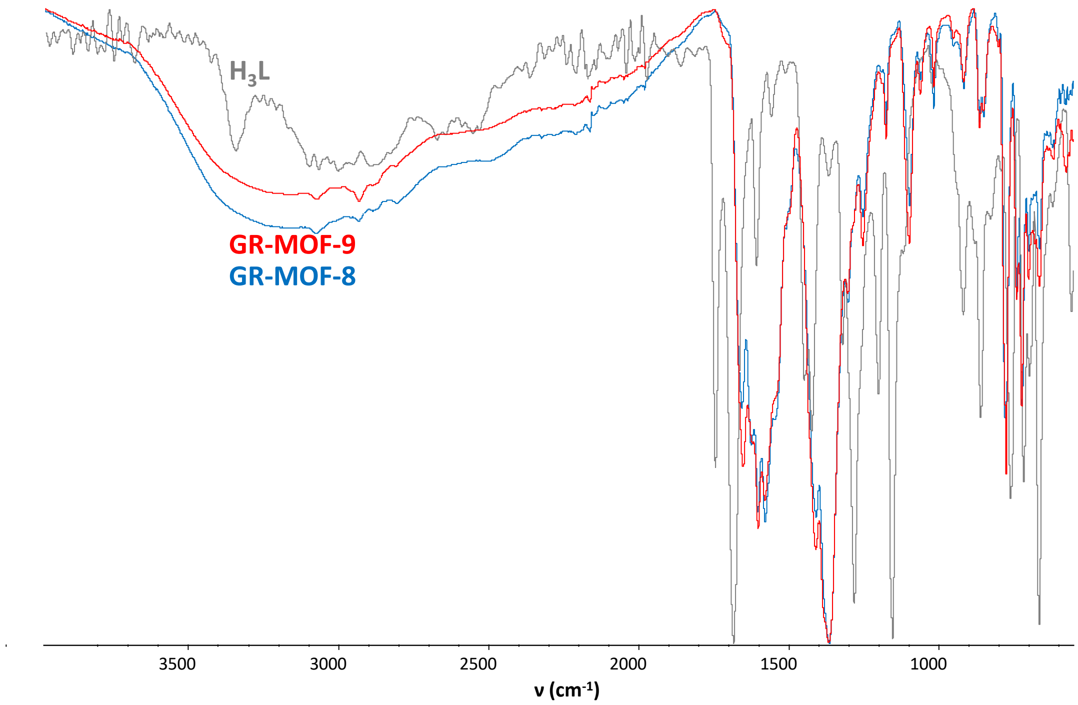

Figure S3. Fourier transform infrared spectroscopy (FTIR) spectra of the GR-MOF-8 (blue) and 9 (red), and the H_3L linker (grey).

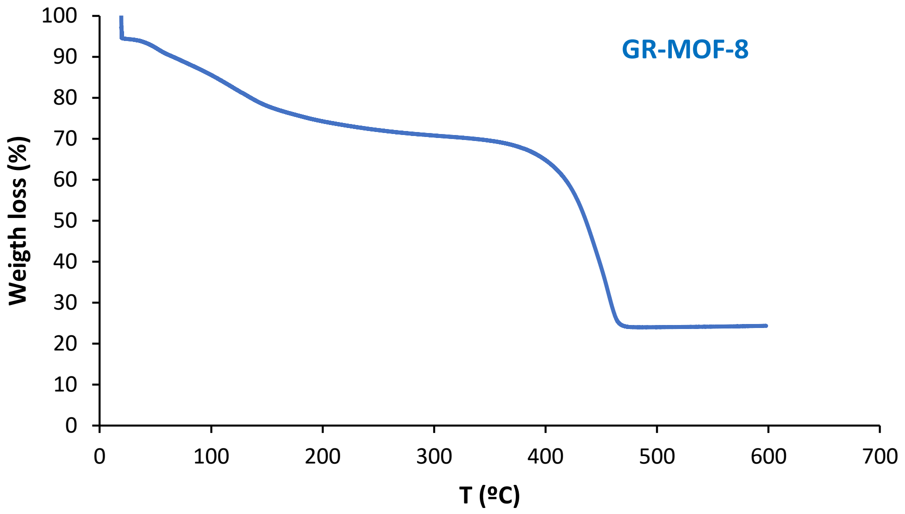

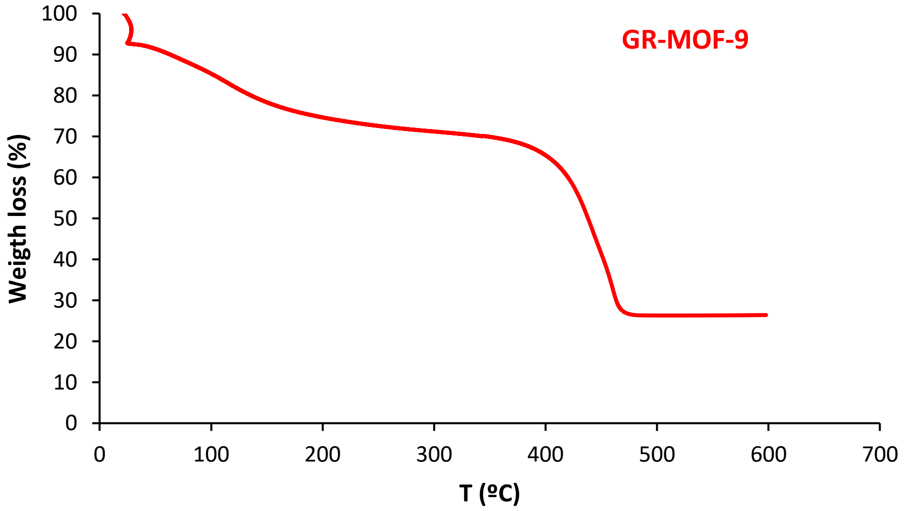

Figure S4. Thermogravimetric analysis (TGA) of the GR-MOF-8 and 9 materials.

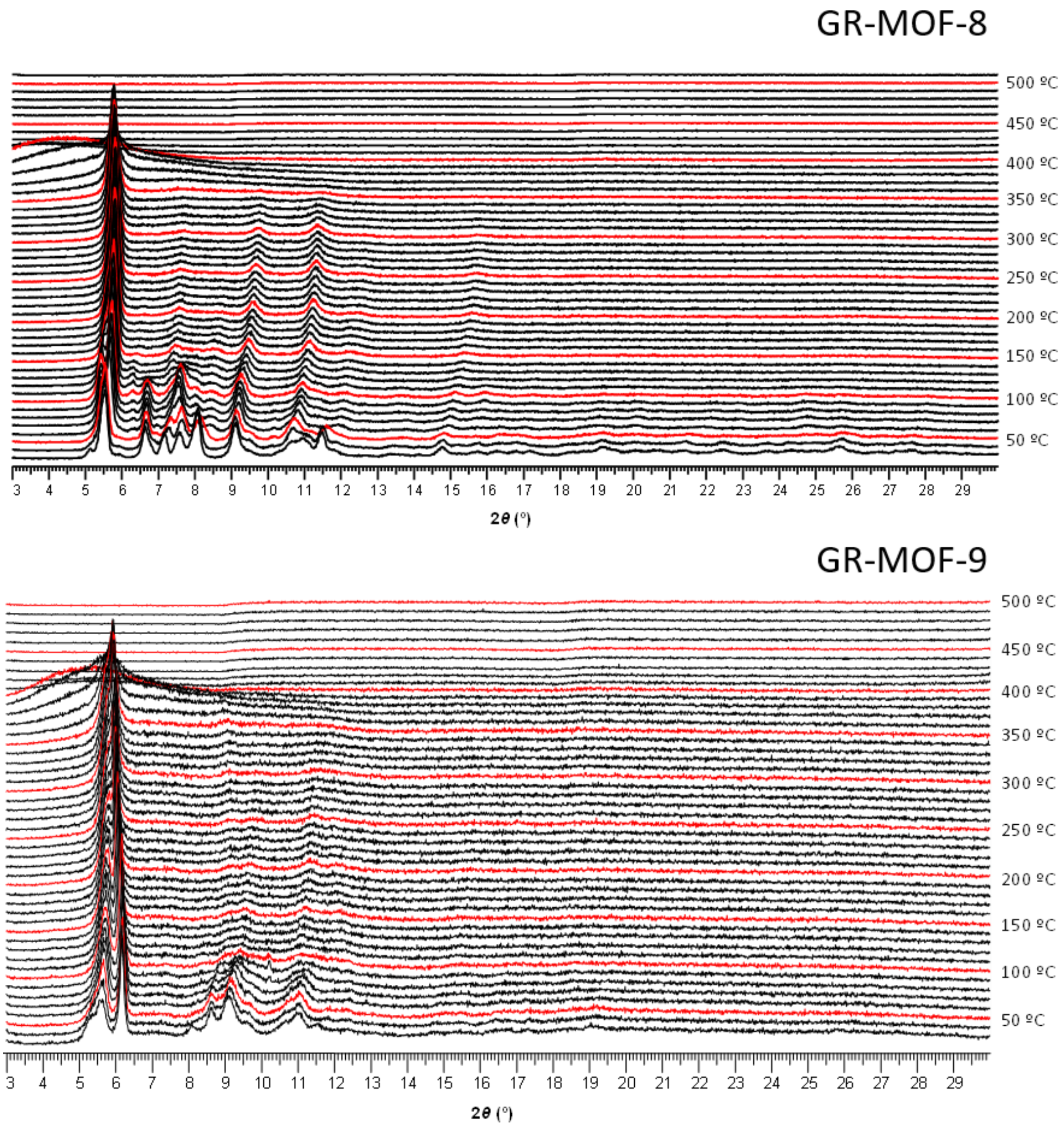

Figure S5. Variable-temperature powder X-ray diffraction (VTPXRD) of the GR-MOF-8 and 9 materials from 25 to 500 °C.

S4. Antibacterial activity

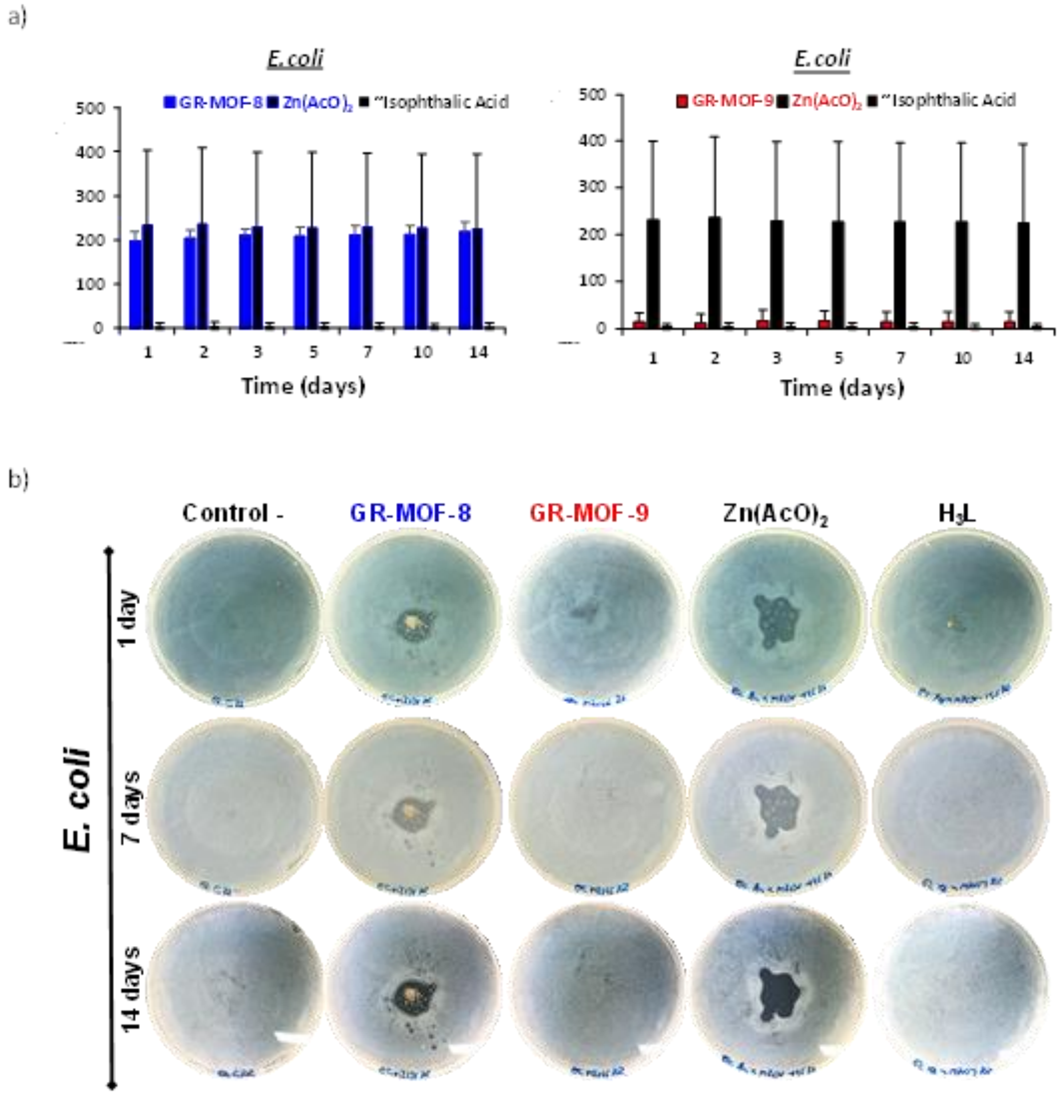

Figure S6. a) Halo inhibition zone experiments (mm²) of GR-MOF-8 (blue), GR-MOF-9 (red) and their precursors: $Zn(AcO)_2{\cdot}H_2O$, H_3L and negative control (C-) after 14 days in contact with *E. coli* cultures in agar plates at 37 °C; **b**) Representative images of inhibition experiments with culture of *E. coli* corresponding to GR-MOF-8 and GR-MOF-9, $Zn(AcO)_2{\cdot}H_2O$, H_3L and negative control in the same conditions.

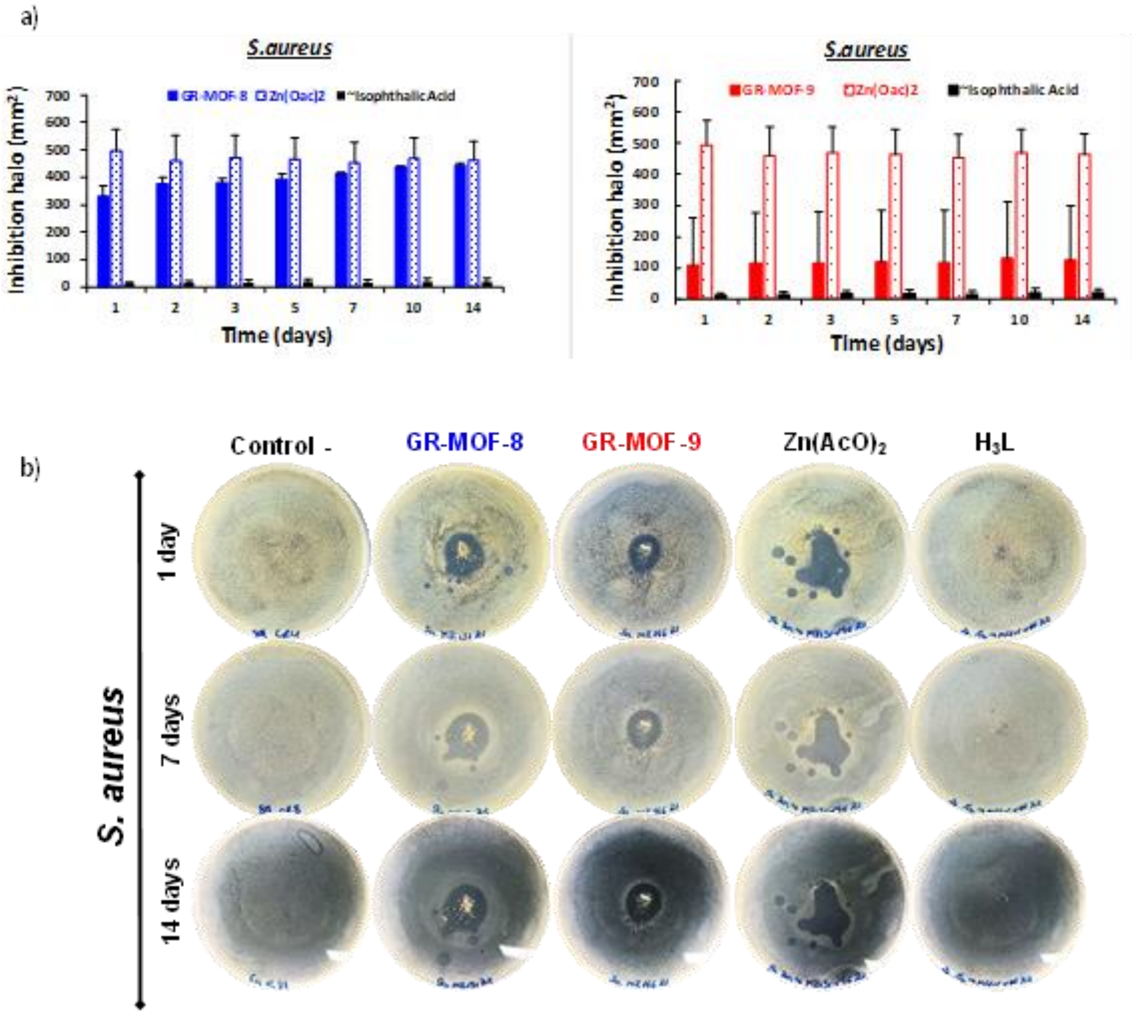

Figure S7. a) Halo inhibition zone experiments (mm^2) of GR.MOF-8 (blue), GR-MOF-9 (red) and their precursors: $Zn(AcO)_2{\cdot}H_2O$, H_3L and negative control (C-) after 14 days in contact with *S. aureus* cultures in agar plates at 37 °C; b) Representative images of inhibition experiments with culture of *S. aureus* corresponding to GR-MOF-8 and GR-MOF-9, $Zn(AcO)_2{\cdot}H_2O$, H_3L, and negative control in the same conditions.

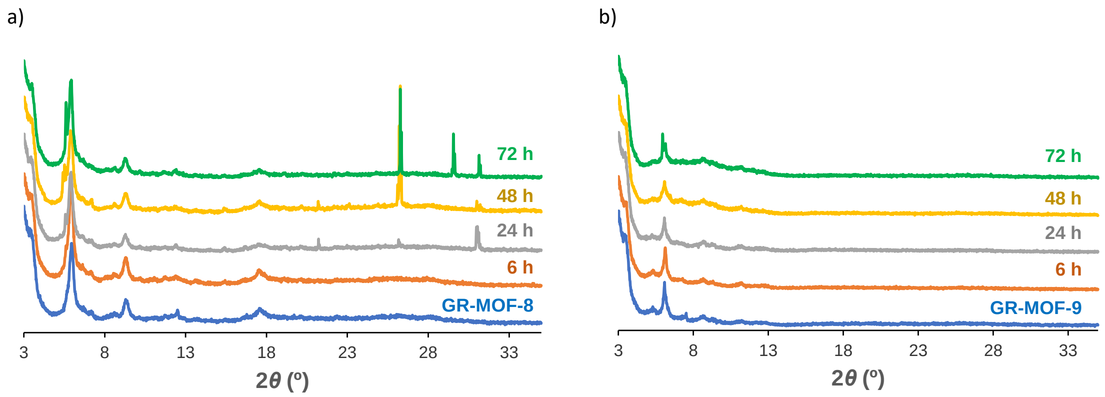

Figure S8. XRP diffraction stability studies of a) GR-MOF-8 and b) GR_MOF-9 in a high relative humidity (70%) ambient.
